# Overexpression of Lrp5 enhanced the anti-breast cancer effects of osteocytes in bone

**DOI:** 10.1038/s41413-021-00152-2

**Published:** 2021-07-06

**Authors:** Shengzhi Liu, Di Wu, Xun Sun, Yao Fan, Rongrong Zha, Aydin Jalali, Yan Feng, Kexin Li, Tomohiko Sano, Nicole Vike, Fangjia Li, Joseph Rispoli, Akihiro Sudo, Jing Liu, Alexander Robling, Harikrishna Nakshatri, Bai-Yan Li, Hiroki Yokota

**Affiliations:** 1 Department of Biomedical Engineering, Indiana University Purdue University Indianapolis, Indianapolis, IN USA; 2 Department of Pharmacology, School of Pharmacy, Harbin Medical University, Harbin, China; 3 Department of Pharmacy, The Second Affiliated Hospital of Dalian Medical University, Dalian, China; 4 Department of Orthopedic Surgery, Mie University, Tsu, Mie Japan; 5 Weldon School of Biomedical Engineering, Purdue University, West Lafayette, IN USA; 6 Department of Physics, Indiana University Purdue University Indianapolis, Indianapolis, IN USA; 7 Department of Anatomy and Cell Biology, Indiana University School of Medicine, Indianapolis, IN USA; 8 Indiana Center for Musculoskeletal Health, Indiana University School of Medicine, Indianapolis, IN USA; 9 Department of Surgery, Indiana University School of Medicine, Indianapolis, IN USA; 10 Simon Cancer Center, Indiana University School of Medicine, Indianapolis, IN USA

**Keywords:** Bone cancer, Bone

## Abstract

Osteocytes are the most abundant cells in bone, which is a frequent site of breast cancer metastasis. Here, we focused on Wnt signaling and evaluated tumor-osteocyte interactions. In animal experiments, mammary tumor cells were inoculated into the mammary fat pad and tibia. The role of Lrp5-mediated Wnt signaling was examined by overexpressing and silencing Lrp5 in osteocytes and establishing a conditional knockout mouse model. The results revealed that administration of osteocytes or their conditioned medium (CM) inhibited tumor progression and osteolysis. Osteocytes overexpressing Lrp5 or β-catenin displayed strikingly elevated tumor-suppressive activity, accompanied by downregulation of tumor-promoting chemokines and upregulation of apoptosis-inducing and tumor-suppressing proteins such as p53. The antitumor effect was also observed with osteocyte-derived CM that was pretreated with a Wnt-activating compound. Notably, silencing Lrp5 in tumors inhibited tumor progression, while silencing Lrp5 in osteocytes in conditional knockout mice promoted tumor progression. Osteocytes exhibited elevated Lrp5 expression in response to tumor cells, implying that osteocytes protect bone through canonical Wnt signaling. Thus, our results suggest that the Lrp5/β-catenin axis activates tumor-promoting signaling in tumor cells but tumor-suppressive signaling in osteocytes. We envision that osteocytes with Wnt activation potentially offer a novel cell-based therapy for breast cancer and osteolytic bone metastasis.

## Introduction

Primary bone cancer is relatively rare, but bone is a frequent site of cancer metastases.^1,2^ Estrogen receptor (ER)-positive cancers preferentially metastasize to bone,^3^ but bone is also a common site of metastasis of ER-negative breast cancers, including triple-negative breast cancer.^4^ Many potential reasons for the high rate of bone metastasis, including chemotaxis mediated by cytokines, chemokines, and TGFβ-rich calcified matrix as a metastatic environment, have been considered.^5^ However, the role of osteocytes, which are the most abundant type of bone cells, in this process remains poorly understood.^6^ Focusing on the role of Wnt signaling, which is required for load-driven bone homeostasis, we evaluated the interactions of tumor cells with mechanosensitive osteocytes.

The extracellular matrix in bone is remodeled by bone-forming osteoblasts and bone-resorbing osteoclasts.^7^ Osteocytes are matrix-laden, differentiated osteoblasts, and mature osteocytes have an elevated level of Sclerostin.^8^ In response to mechanical stimulation, these cells activate Wnt signaling and orchestrate bone remodeling.^9^ With their extensive dendritic architecture, osteocytes act as mechanosensors.^10^ Low-density lipoprotein receptor-related protein 5 (Lrp5) is a Wnt coreceptor^11^ that promotes loading-driven bone formation by downregulating Sclerostin.^7^ In the canonical Wnt signaling pathway, β-catenin acts as an epicenter of intracellular signal transduction.^12^ In contrast to its beneficial role in osteocytes, Wnt signaling plays a detrimental role in cancer, and thus strategies to inhibit Wnt signaling have been sought.^13^ This study aimed to examine the role of Lrp5- and β-catenin-mediated Wnt signaling in tumor-osteocyte interactions in order to develop a novel therapeutic strategy based on Wnt regulation. To the best of our knowledge, the effect of osteocytic Wnt activation on tumor-osteocyte interactions has not been investigated.

Instead of direct application of a Wnt-modulating agent to tumor cells, our approach in this study was to activate Wnt signaling in cultured osteocytes and apply these osteocytes with Wnt activation or conditioned medium (CM) from these cultures to tumors. Cell-based therapies are being attempted for various diseases due to recent technological advances.^14^ In regenerative medicine, CM derived from mesenchymal stem cells has been employed to promote the healing of damaged tissues.^15^ However, the effects of osteocyte-derived CM on breast cancer-associated osteolysis have not been well studied. In this study, we first overexpressed Wnt-modulating genes, such as Lrp5 and β-catenin, in osteocytes or pretreated osteocytes with the chemical agent BML284, a Wnt activator. The pretreated osteocytes or their CM were then administered locally or systemically in a mouse model of breast cancer as well as a mouse model of tumor-induced tibial osteolysis.

Tumor heterogeneity results from complex cellular interactions, through which the growth of tumor cells can be promoted or inhibited depending on the neighboring tumor and nontumor cells.^16^ In this study, we mainly employed differentiated MLO-A5 osteocytes that had an elevated expression level of Sclerostin. It has been reported that peripheral blood and bone marrow plasma from multiple myeloma patients contain elevated levels of Sclerostin.^17^ Additionally, an anti-Sclerostin antibody was shown to increase bone mass without affecting the progression of the tumor.^18^ However, we observed herein that mature osteocyte-derived CM had a stronger tumor-suppressive capability than premature osteocyte-derived CM, and the results indicated a dichotomous role for Sclerostin as well as Lrp5 in osteocytes and tumor cells. Collectively, our results reveal opposing roles of the Lrp5/β-catenin axis in tumor cells and osteocytes; these roles impact the metastatic progression of cancer and potentially provide an explanation for the limited success in targeting this signaling axis in advanced cancers.

## Results

### Differentiated osteocytes inhibited the proliferation, migration, and invasion of mammary tumor cells

**Fig. 1:**
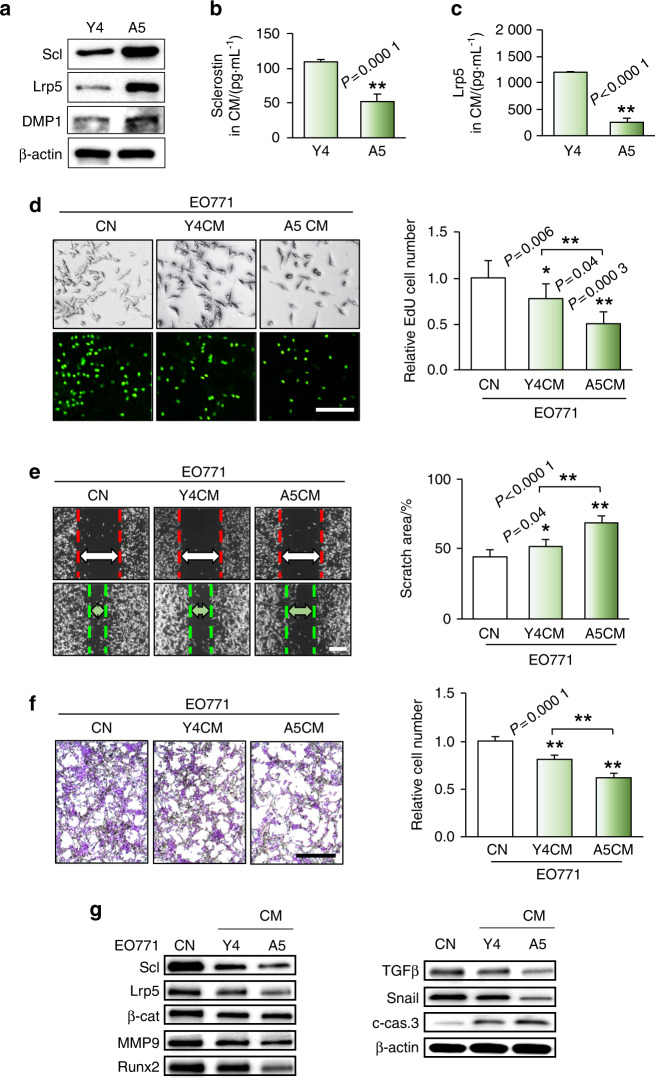
Osteocyte-driven inhibition of tumorigenic behaviors of EO771 mammary tumor cells. CN control (plain medium), CM conditioned medium, A5 differentiated MLO-A5 osteocytes, Y4 MLO-Y4 osteocytes, Scl sclerostin, and β-cat β-catenin. The single and double asterisks indicate *P* < 0.05 and *P* < 0.01, respectively. **a** Elevated levels of Sclerostin (Scl), Lrp5, and DMP1 in differentiated MLO-A5 osteocytes compared to MLO-Y4 osteocytes. **b**, **c** Lower levels of Scl and Lrp5 in A5 CM than in Y4 CM. **d**–**f** Significant reductions in the proliferation (as assessed by an EdU incorporation assay), migration (as assessed by a wound-healing assay), and invasion (as assessed by a Transwell assay) of EO771 mammary tumor cells by Y4 CM and A5 CM compared to control (plain medium). Scale bar: 200 μm. **g** Reductions in the levels of Scl, Lrp5, β-catenin, MMP9, Runx2, TGFβ, and Snail with an increase in cleaved caspase 3 in EO771 cells in response to Y4 CM and A5 CM

To evaluate the role of osteocytes in cancer progression, we first differentiated MLO-A5 preosteocytes by treating them with ascorbic acid. Compared to MLO-Y4 osteocyte-like cells, which are premature osteocytes, the ascorbic acid-treated A5 osteocytes themselves expressed elevated levels of Sclerostin (Scl), a marker of osteocyte differentiation and maturity; Lrp5, a coreceptor for Wnt signaling; and DMP1, a matrix protein involved in bone mineralization (Fig. 1a). By contrast, we observed that A5 osteocyte-derived CM contained lower levels of Sclerostin and Lrp5 than Y4 osteocyte-derived CM (Fig. 1b, c). Compared to Y4 CM, A5 CM exhibited stronger inhibitory effects on the proliferation, migration, and invasion of EO771 mammary tumor cells, based on the results of EdU incorporation assays, wound-healing assays, and Transwell assays, respectively (Fig. 1d–f). Furthermore, we evaluated the expression of tumor-promoting genes that play critical roles in the progression and metastasis of breast cancer, such as MMP9, Runx2, TGFβ, and Snail. The western blot results showed that in EO771 cells, A5 CM downregulated tumor-promoting genes such as Sclerostin, Lrp5, β-catenin, MMP9, Runx2, TGFβ, and Snail and elevated the level of an apoptosis marker, cleaved caspase 3, more substantially than did Y4 CM (Fig. 1g). The above results indicated that A5 osteocyte-derived CM, containing a low level of Sclerostin, did not contribute to tumor progression but inhibited the proliferation, migration, and invasion of tumor cells.

### Differentiated osteocytes inhibited migration and invasion in vitro, ex vivo, and in vivo

**Fig. 2:**
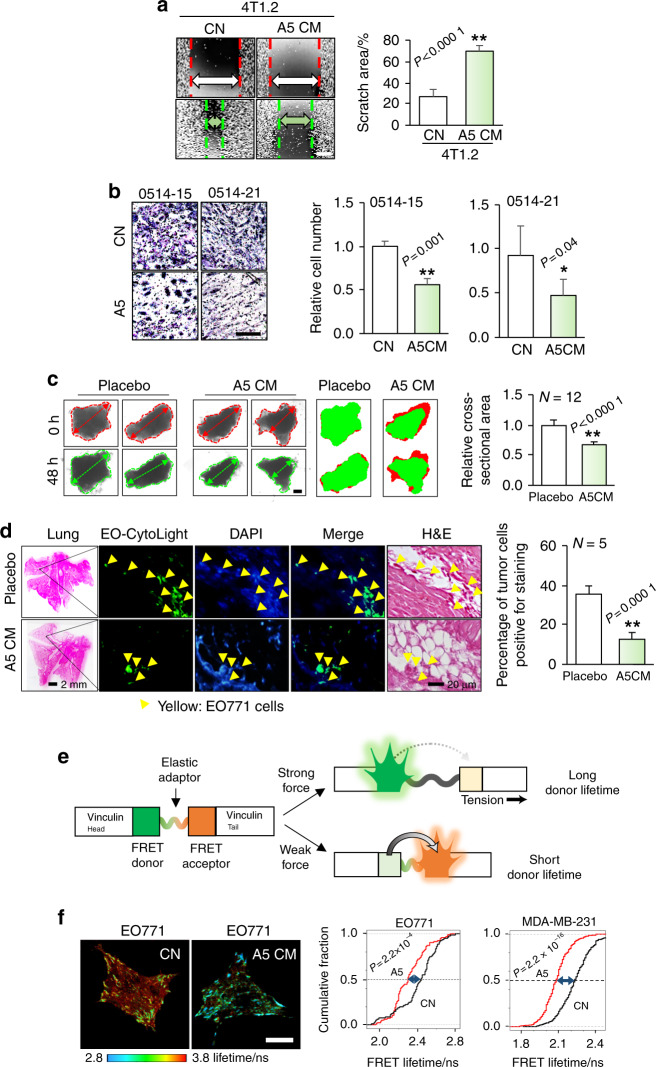
Osteocyte-driven tumor-suppressive capabilities in vitro, ex vivo, and in vivo. CN control (plain medium), and CM conditioned medium. The single and double asterisks indicate *P* < 0.05 and *P* < 0.01, respectively. **a** Reduction in the migration (as assessed by a wound-healing assay) of 4T1.2 mammary tumor cells induced by A5 CM. Scale bar: 200 μm. **b** Significant reduction in the invasion of primary human breast cancer cells from 2 sources after 48 h of treatment with A5 osteocyte-derived CM. Scale bar: 200 μm. **c** Significant shrinkage of ex vivo breast cancer tissue fragments by A5 osteocyte-derived CM. The green image at 48 h is overlaid on the red image at 0 h. Scale bar: 200 μm. **d** Significant reduction in the number of green fluorescence-labeled EO771 mammary tumor cells (yellow arrows) in the lungs of C57BL/6 mice after coinjection with A5 osteocyte-derived CM in the extravasation assay. **e** Schematic diagram of a vinculin FRET biosensor: the tension-sensitive sensor consists of the head and tail domains of vinculin with an elastic FRET module. In response to a strong (or weak) force, the sensor exhibits a long (or short) donor lifetime. **f** A5 CM-driven significant reduction in the fluorescence lifetime of a vinculin biosensor in EO771 and MDA-MB-231 cells. The observed reduction in the fluorescence lifetime indicates A5 CM’s inhibitory effects on molecular force and cell migration. Scale bar: 20 μm

We next determined the effects of CM from differentiated osteocytes on the migratory and invasive properties of mammary tumor cells. A5 CM reduced the migration of 4T1.2 mammary tumor cells, as evaluated by a wound-healing assay (Fig. 2a), and the invasion of primary human breast cancer cells, as evaluated by a Transwell assay (Fig. 2b). Of note, 0514-15 cells were derived from a pleural effusion in a patient with ER^+^/PR^−^ breast cancer, whereas 0514-21 cells were derived from a chest wall metastasis in a patient with triple-negative breast cancer. A5 CM also inhibited the ex vivo growth of human breast cancer tissue fragments (Fig. 2c) and reduced the number of colonized tumor cells in the mouse lung in the in vivo extravasation assay (Fig. 2d). To examine the effect of osteocytes at the biophysical level, we employed a vinculin tension sensor. The vinculin head and tail domains were linked to a FRET donor and acceptor, respectively^19^ (Fig. 2e). We determined the fluorescence lifetime of the FRET donor. Compared to the control medium, A5 CM shortened the FRET donor lifetime in EO771 and MDA-MB-231 cells (Fig. 2f), indicating that A5 CM weakened tensile forces at focal adhesions.

### Lrp5, expressed in osteocytes, enhanced antitumor capability in vitro

**Fig. 3:**
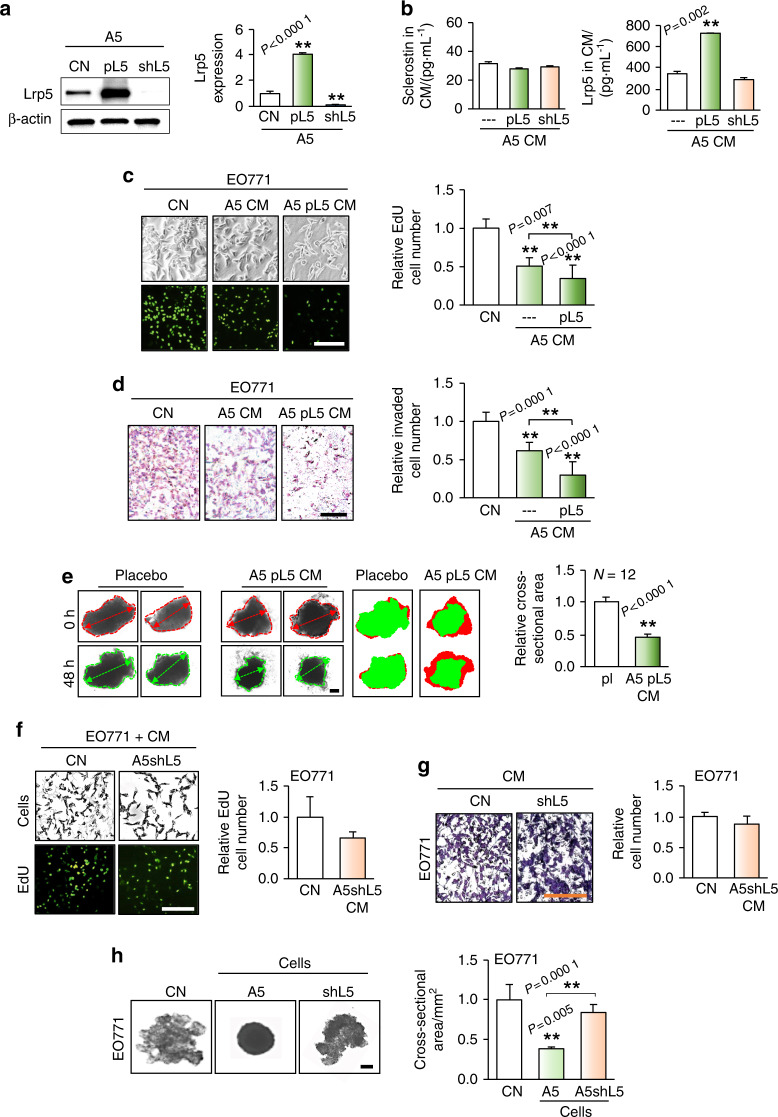
Enhancement of tumor-suppressive capability by overexpression of Lrp5 in A5 osteocytes. CN control, CM conditioned medium, pL5 Lrp5 plasmids, and shL5 Lrp5 shRNA. The single and double asterisks indicate *P* < 0.05 and *P* < 0.01, respectively. **a** Alteration in the Lrp5 level in A5 osteocytes by overexpression and silencing of Lrp5. **b** Alterations in the levels of Sclerostin and Lrp5 in A5 CM in response to overexpression and silencing of Lrp5 in A5 osteocytes. **c**, **d** Enhanced reductions in the proliferation (as assessed by an EdU incorporation assay) and invasion (as assessed by a Transwell assay) of EO771 cells in response to treatment with CM from Lrp5-overexpressing A5 cells. Scale bar: 200 μm. **e** Significant shrinkage of ex vivo breast cancer tissue fragments induced by treatment with CM from Lrp5-overexpressing A5 cells. The green image at 48 h is overlaid on the red image at 0 h. Scale bar: 200 μm. **f**–**h** Suppression of the antitumor action of A5 CM by silencing Lrp5 in A5 osteocytes, ass assessed by EdU-based proliferation, Transwell invasion, and tumor spheroid assays using EO771 mammary tumor cells. Scale bar: 200 μm

The above results suggested the antitumor action of osteocytes. Since osteocytes are known to regulate Wnt signaling, we next examined whether overexpression of Lrp5 in osteocytes alters their antitumor capability (Fig. 3a). Overexpression of Lrp5 in A5 osteocytes elevated the level of Lrp5 in A5 osteocytes and their CM, while silencing of Lrp5 reduced its level in A5 osteocytes (Fig. 3a, b). Of note, the level of Sclerostin in A5 CM was not altered by either overexpression or silencing of Lrp5 (Fig. 3b). We observed that Lrp5-overexpressing osteocyte-derived CM inhibited the proliferation and invasion of EO771 tumor cells, as determined by EdU incorporation and Transwell assays, respectively (Fig. 3c, d). Furthermore, Lrp5-overexpressing osteocyte-derived CM significantly inhibited the ex vivo growth of cancer tissue fragments (Fig. 3e). By contrast, Lrp5-silenced osteocyte-derived CM lost the ability to inhibit cell proliferation and invasion (Fig. 3f, g), as well as the growth of tumor spheroids (Fig. 3h). Collectively, these results indicated that Lrp5 in osteocytes was partially responsible for their antitumor action.

Enhancement of antitumor effects by overexpression of Lrp5 was also observed in cells treated with Y4 CM (Supplementary Fig. 1a, b). However, this antitumor function of Lrp5 was not observed in cells treated with fibroblast-derived CM (Supplementary Fig. 1c–e). We observed that CM from human osteocytes either with or without Lrp5 overexpression also inhibited the proliferation, invasion, and migration of tumor cells (Supplementary Fig. 2a). Furthermore, CM from Lrp5-overexpressing but not parental osteocytes reduced the proliferative and invasive properties of PC-3 prostate cancer cells (Supplementary Fig. 2b, c). Taken together, these results indicated that Lrp5-overexpressing mouse and human osteocytes acted as tumor suppressors not only in breast cancer cells but also in prostate cancer cells.

### A5 osteocytes reduced mammary tumor growth in vivo

Using a proof-of-principle model with C57BL6 mice, we next evaluated the effect of osteocytes on mammary tumors, which provided preclinical proof of principle of osteocytes’ antitumor efficacy. EO771 cells were injected into the seventh mammary fat pad with and without coinjection of osteocytes. Coinjection of osteocytes reduced the growth of mammary tumors (Supplementary Fig. 3a). Examination of a histological section containing GFP-labeled osteocytes showed that osteocytes were mostly located in the proliferative zone, where they were interfused with tumor tissue (Supplementary Fig. 3b). We also observed the same tumor-suppressive effect in NOD/SCID mice injected with MDA-MB-231 breast cancer cells (Supplementary Fig. 3c, d). The effect of Lrp6, another coreceptor in Wnt signaling, was different from that of Lrp5. Injection of Lrp6-silenced EO771 cells did not alter mammary tumors (Supplementary Fig. 3e, f), and Lrp6-silenced osteocyte-derived CM did not suppress the antitumor action of osteocytes (Supplementary Fig. 3g, h). Collectively, these results indicated that Lrp5 but not Lrp6 altered the tumor-suppressive capability of osteocytes.

### Osteocyte coinjection suppressed tumor-induced osteolysis

**Fig. 4:**
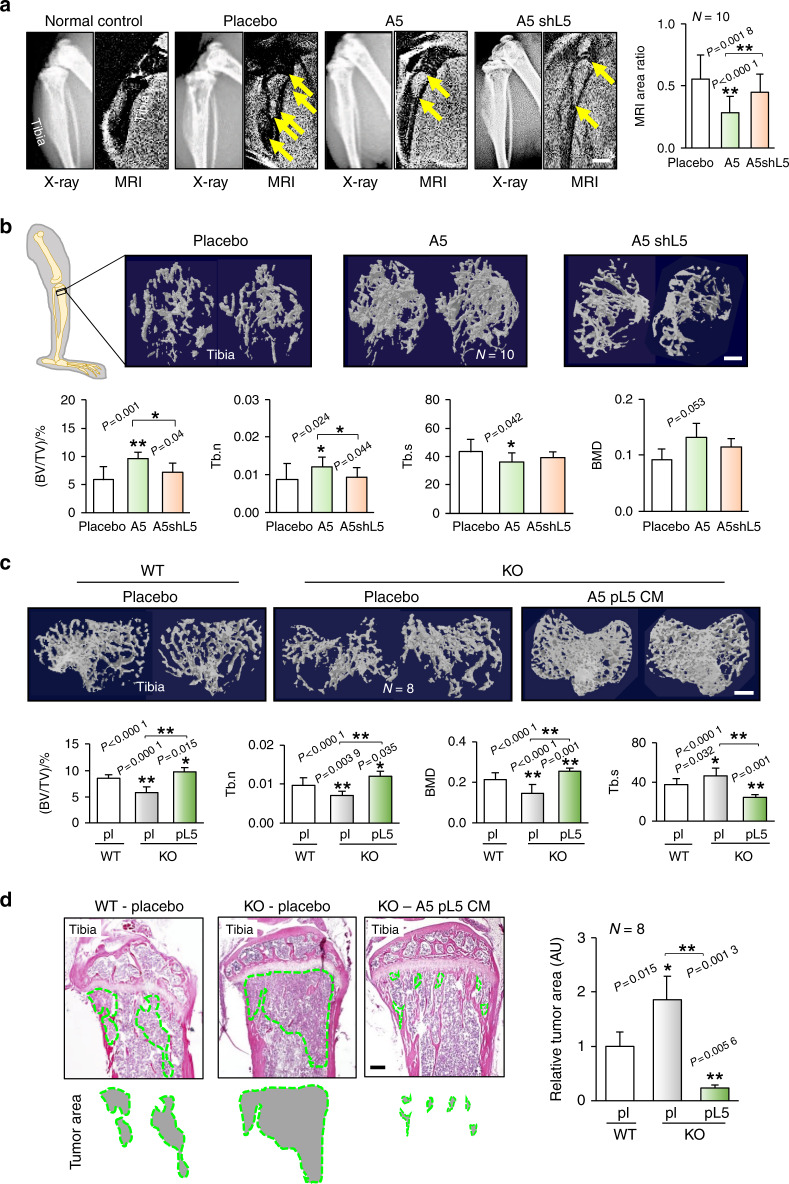
Effects of coinjection of A5 osteocytes and tumor cells into the tibia in C57BL/6 mice. pl placebo, shL5 Lrp5 shRNA, pL5 Lrp5 plasmids, WT wild-type, KO Lrp5 conditional knockout, and CM conditioned medium. The single and double asterisks indicate *P* < 0.05 and *P* < 0.01, respectively. **a** Representative X-ray and MR images of the tibia in the normal control (healthy group), placebo, A5 cell-coinjected, and Lrp5 shRNA-treated A5 groups. The yellow arrow in the MR image indicates a tumor-induced lesion in the tibia. The MR image-based percentage area of the tumor-induced lesion is shown. Scale bar: 1 mm. **b** Representative μCT images of the proximal tibia in the placebo, A5 cell-coinjected, and Lrp5 shRNA-treated A5 groups. μCT-based parameters are shown: BV/TV bone volume normalized by total volume, Tb.N trabecular number, Tb.S trabecular separation, and BMD bone mineral density. Scale bar: 500 μm. **c** Representative μCT images of the proximal tibia, showing the severe bone loss caused by tumor growth in Lrp5 KO mice and its rescue by Lrp5-overexpressing osteocyte-derived CM. Scale bar: 500 μm. **d** H&E-stained proximal tibiae of mice with Lrp5 KO. Lrp5-overexpressing osteocyte-derived CM significantly reduced tumor growth. The green-shaded area shows the tumor-invaded region. Of note, tumor cells typically appeared hyperchromatic with an increased nucleoplasmic ratio. Scale bar: 200 μm

Thus far, we have shown that osteocytes act as tumor suppressors in the mammary fat pad. We next examined their effect on tumor invasion of bone. MR imaging showed that mice in the placebo control group (injected with only tumor cells) exhibited clear tumor-linked lesions in the tibia, which were significantly reduced in the A5-injected group (Fig. 4a). Furthermore, μCT imaging revealed that osteocyte coinjection reduced tumor-driven osteolysis (Fig. 4b). In response to the coinjection, the bone volume ratio (BV/TV) and trabecular number (Tb.N) were elevated, and the trabecular separation (Tb.S) was reduced. These changes suggest the ability of osteocytes to protect against cancer-induced osteolysis. No benefits of osteocyte coinjection were observed when osteocytes expressing Lrp5 shRNA were used. Evaluation of H&E-stained sagittal sections revealed that the osteocyte-injected group exhibited reduced tumor growth and bone injury (Supplementary Fig. 4a). These results indicate that Lrp5-positive osteocytes contribute to the prevention of tumor-induced bone loss. Of note, Lrp5 expressed in tumor cells has a protumorigenic role, as mice injected with Lrp5-silenced EO771 tumor cells showed a reduction in mammary tumor growth and tumor-driven bone loss (Supplementary Fig. 4b, c).

### Lrp5 deletion in osteocytes worsened tumor-driven osteolysis in vivo

Having shown Lrp5’s antitumor capability in osteocytes, we next examined the effect of Lrp5 deletion in osteocytes on tumor progression in the tibia in conditional knockout mice. As predicted, mice with osteocytic deletion of Lrp5 exhibited significantly lower bone mass than their wild-type littermates (Fig. 4c, d). However, local injection of Lrp5-overexpressing osteocyte-derived CM into the proximal tibia markedly protected the bone. In addition to the tumor-driven reduction in BV/TV, we also observed a reduction in BV/TV by deletion of Lrp5 in osteocytes (Supplementary Fig. 4d). However, the tumor-induced reduction in BV/TV was greater than that caused by deletion of Lrp5 in osteocytes (Supplementary Fig. 4e).

### Lrp5 in osteocytes downregulated tumor-promoting genes and upregulated tumor-suppressive genes

**Fig. 5:**
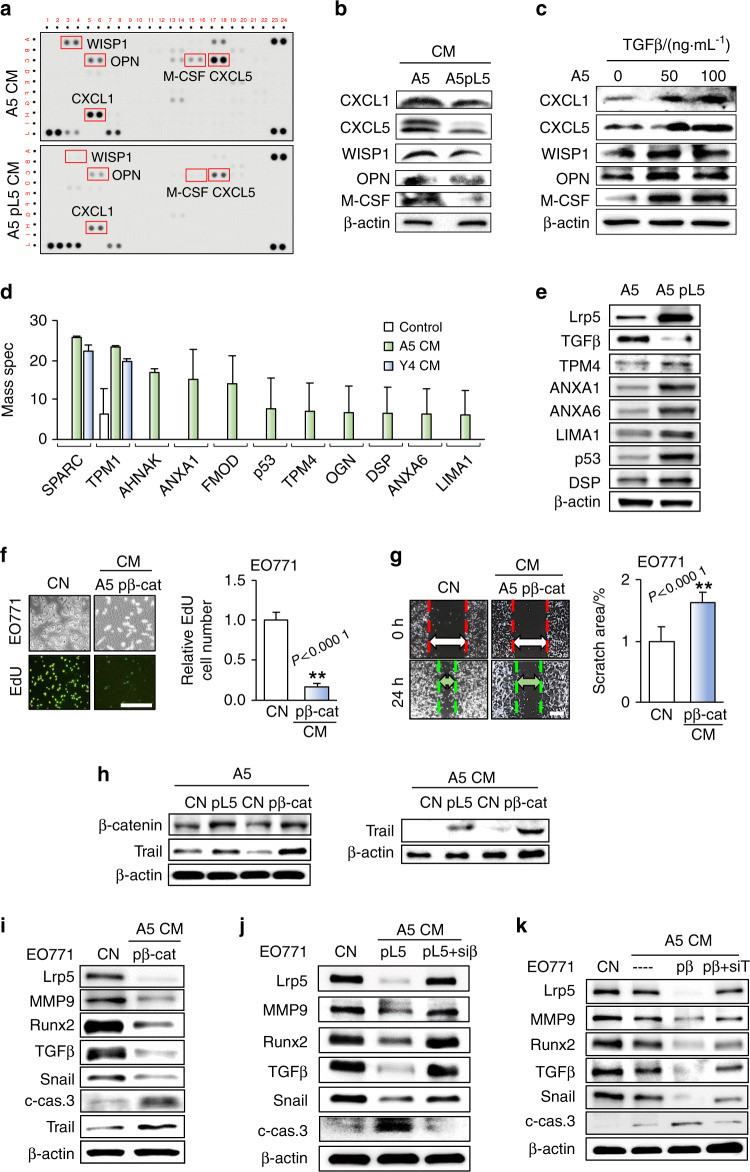
Regulation of tumor-promoting and tumor suppressor genes by A5 osteoblasts. CN control (plain medium), CM conditioned medium, pL5 Lrp5 plasmids, pβ-cat β-catenin plasmids, siβ β-catenin siRNA, and siT Trail siRNA. **a** Comparison of the expression levels of 111 cytokines in CM from parental A5 cells and CM from Lrp5-overexpressing A5 cells. The levels of CXCL1 and CXCL5 were decreased in CM from Lrp5-overexpressing osteocytes. **b** Expression of CXCL1, CXCL5, WISP1, OPN, and M-CSF in A5 osteocytes with and without Lrp5 overexpression. **c** Elevation of CXCL1, CXCL5, WISP1, OPN, and M-CSF by TGFβ. **d** Proteins enriched in A5 osteocyte-derived CM. **e** Expression of TGFβ, TPM4, ANXA1, ANXA6, LIMA1, p53, and DSP in A5 osteocytes with and without Lrp5 overexpression. **f**, **g** Significant reductions in the proliferation and migration of EO771 cells after treatment for 48 h with β-catenin-overexpressing osteocyte-derived CM. Scale bar: 200 μm. **h** Elevation of β-catenin and Trail by overexpression of Lrp5 and β-catenin in A5 osteocytes and in their conditioned medium. **i** Protein levels of Lrp5, MMP9, Runx2, TGFβ, Snail, cleaved caspase 3, and Trail in EO771 cells in response to β-catenin-overexpressing osteocyte-derived CM. **j** Suppression of the inhibitory effects of Lrp5-overexpressing osteocyte-derived CM by silencing of β-catenin. **k** Suppression of the inhibitory effects of β-catenin-overexpressing osteocyte-derived CM by silencing of Trail

To understand the regulatory mechanism of Lrp5’s action, analysis of a 111-cytokine antibody array was conducted with CM from osteocytes with and without Lrp5 overexpression. In Lrp5-overexpressing CM, the levels of two chemokine ligands (CXCL1 and CXCL5) and three other proteins (WISP1, OPN, and M-CSF) were significantly reduced (Fig. 5a, b; Supplementary Fig. 5a). These chemokines/cytokines are protumorigenic and prometastatic, and at least two of the chemokines (CXCL1 and CXCL5) are the targets of the prometastatic cytokine TGFβ in osteocytes (Fig. 5c). In addition to downregulating tumor-promoting genes, osteocyte-derived CM was enriched with proteins that potentially acted as tumor suppressors. Mass spectrometry-based analysis predicted that the levels of TPM4, ANXA1, ANXA6, LIMA1, p53, and DSP were elevated in A5 osteocyte-derived CM (Fig. 5d). In Lrp5-overexpressing osteocytes, the expression levels of these genes were elevated, but that of TGFβ was reduced (Fig. 5e). The Lrp5 overexpression-induced changes in the expression levels of these proteins in osteocyte CM could contribute to the inhibition of tumor progression.

### Overexpression of β-catenin and the treatment with the Wnt activator BML284 enhanced the antitumor capability of osteocytes

Since Lrp5 is involved in Wnt signaling, we examined the status of β-catenin, which is a downstream mediator of Wnt signaling. We observed that in EO771 cells, β-catenin-overexpressing CM elevated apoptosis-linked genes (CYCS, HIF1α, and APT1) in EO771 cells (Supplementary Fig. 5b). The EdU incorporation assay and Transwell-based invasion assay revealed that β-catenin-overexpressing osteocyte-derived CM reduced the proliferation and migration of EO771 tumor cells (Fig. 5f, g). In osteocytes, overexpression of Lrp5 increased the expression of β-catenin and an apoptosis-inducing factor, Trail (Fig. 5h), while it downregulated tumorigenic proteins such as MMP9, Runx2, TGFβ, and Snail (Fig. 5i). By contrast, silencing of β-catenin reversed the expression profile of osteocytes with overexpression of Lrp5 (Fig. 5j; Supplementary Fig. 5c). Notably, the inhibitory effect of CM from β-catenin-overexpressing osteocytes on tumor-promoting proteins was suppressed by silencing of Trail (Fig. 5k).

We determined the levels of Sclerostin and Lrp5 in CM from β-catenin-overexpressing osteocytes and osteocytes treated with BML284, an activator of Wnt signaling. The levels of these proteins were not elevated in either CM. Instead, β-catenin-overexpressing CM exhibited a decreased Sclerostin level, and BML284-treated CM exhibited a decreased Lrp5 level (Supplementary Fig. 5d). BML284-treated CM enhanced the antitumor capability of osteocytes in an ex vivo tissue assay (Supplementary Fig. 5e). It also downregulated tumor-promoting genes, and its administration to C57BL/6 mice reduced the progression of tumors (Supplementary Fig. 5f–h).

Of note, the levels of tumor-suppressive genes (TPM4, ANXA1, ANXA6, LIMA1, p53, and DSP) were elevated in β-catenin-overexpressing osteocytes, and CM from β-catenin-overexpressing osteocytes reduced the levels of CXCL1, CXCL5, WISP1, OPN, and M-CSF (Supplementary Fig. 6a, b). Additionally, the levels of tumor-promoting genes (Lrp5, MMP9, Runx2, and Snail) were increased by TGFβ and CXCL5, while they were reduced by TPM4, ANXA6, and Trail (Supplementary Fig. 6c, d). Regarding the link between β-catenin and Trail, CM from β-catenin-overexpressing osteocytes inhibited the proliferation and invasion of tumor cells, but RNA interference with Trail siRNA blocked the inhibitory effects of CM from β-catenin-overexpressing osteocytes (Supplementary Fig. 6e, f).

### CM from β-catenin-overexpressing osteocytes inhibited tumor progression and osteoclastogenesis

**Fig. 6:**
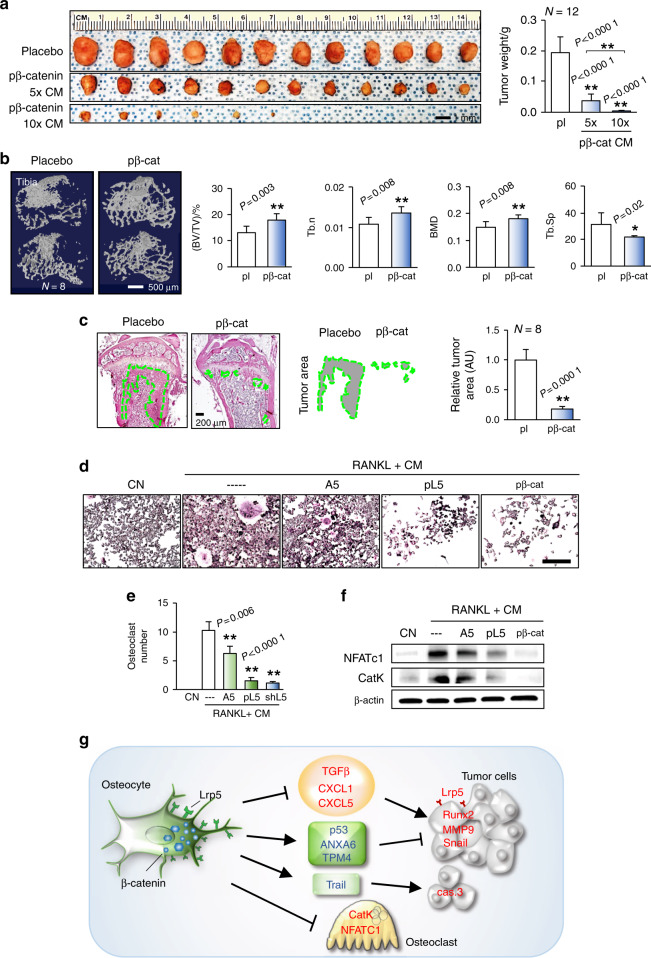
Effects of β-catenin-linked Wnt signaling in A5 osteocytes on tumor progression. CN control (plain medium), CM conditioned medium, pl placebo, pL5 Lrp5 plasmids, and pβ-cat β-catenin plasmids. The single and double asterisks indicate *P* < 0.05 and *P* < 0.01, respectively. **a**–**c** Significant reductions in mammary tumor growth and bone degradation in the tibia in C57BL/6 mice by systemic administration of β-catenin-overexpressing osteocyte-derived CM. **d**–**f** Significant reductions in the number of TRAP-stained mature osteoclasts and the expression of NFATc1 and Cathepsin K in RANKL-stimulated osteoclasts by treatment with Lrp5- and β-catenin-overexpressing osteocyte-derived CM. Scale bar: 200 μm. **g** Schematic illustration of the mechanism regulating tumor-osteocyte interactions. In this diagram, Lrp5 and β-catenin in osteocytes inhibit tumor progression by decreasing the expression of tumor-promoting genes (TGFβ, CXCL1, and CXCL5) and increasing the expression of tumor-suppressing genes (p53, ANXA6, and TPM4) and apoptosis-inducing genes (Trail). In tumor cells, tumor-promoting genes (Lrp5, Runx2, MMP9, and Snail) were downregulated. Overexpression of Lrp5 and β-catenin also inhibited osteoclastogenesis by downregulating NFATc1 and cathepsin K

We observed that systemic administration of β-catenin-overexpressing osteocyte-derived CM inhibited the progression of mammary tumors (Fig. 6a) and tumor-driven osteolysis (Fig. 6b, c, Supplementary Fig. 6g). In addition, we examined the potential involvement of Runx2 in tumor progression. In human primary breast cancer cells from two sources, A5 CM reduced the Runx2 and MMP9 levels (Supplementary Fig. 7a). FRET analysis revealed that Runx2-silenced EO771 cells exhibited a decreased fluorescence lifetime, indicating that silencing of Runx2 reduced the molecular force and cell migration (Supplementary Fig. 7b). Furthermore, in a mouse model, mice inoculated with Runx2-silenced EO771 cells exhibited reduced tumor weights (Supplementary Fig. 7c). To examine the effects of osteocyte-derived CM on the development of bone-resorbing osteoclasts, we performed staining for tartrate-resistant acid phosphatase (TRAP), a marker of osteoclasts. Notably, Lrp5- and CM from β-catenin-overexpressing osteocytes reduced the number of mature osteoclasts, with TRAP-positive multinucleated (>3 nuclei) cells counted as mature osteoclasts (Fig. 6d, e). CM from Lrp5- and β-catenin-overexpressing osteocytes downregulated NFATc1, a master transcription factor for osteoclastogenesis, and cathepsin K, a protease for bone resorption (Fig. 6f).

## Discussion

In this study, we showed that osteocytes or their CM acted as antitumor agents against mammary tumors and suppressed tumor growth in bone in a mouse model. μCT images showed the protection of trabecular bone, and MR images and histological results indicated a reduction in the tumor-invaded region. Ex vivo and in vitro studies revealed the inhibitory effects of osteocyte-derived CM on tumor cell proliferation, migration, and invasion. A FRET vinculin biosensor assay demonstrated that the molecular force in tumor cells was weakened by osteocyte-derived CM. Notably, the Wnt coreceptor Lrp5 and β-catenin played a critical role in osteocyte-driven tumor inhibition. Proof-of-principle studies, in which osteocytes were coinjected with tumor cells into mammary fat pads, verified that osteocytes were potentially able to suppress tumor progression. Systemic administration of CM from osteocytes with overexpression of Lrp5 or β-catenin strikingly reduced mammary tumor growth and tumor-induced bone loss. Enhancement of the antitumor effects was also observed in osteocytes preconditioned with BML284, an activator of Wnt signaling. Collectively, these results indicate that osteocytes and their CM are capable of inhibiting tumor cells by activating endogenous Wnt signaling and inactivating Wnt signaling in invading cancer cells.

Overexpression of Lrp5 and constitutive activation of β-catenin in osteocytes contributed to their enhanced antitumor capability through four distinct mechanisms (Fig. 6g). The first mechanism was downregulation of tumor-promoting genes such as CXCL1, CXCL5, and WISP1,^19–21^ while the second was upregulation of tumor suppressor genes such as p53, ANXA6, and TMP4. Of note, p53 is known as a guardian of the genome and a suppressor of inflammatory responses in the tumor microenvironment.^22^ While p53 is reported to be present in circulating blood and to affect the growth of tumor cells,^23^ the tumor suppressor candidates identified by mass spectrometry are known to mainly function intracellularly. It is thus important to further examine the roles of these candidate genes in CM. The third mechanism was elevation of Trail and induction of apoptosis, and the fourth was suppression of osteoclastogenesis via inhibition of both TRAP-positive multinucleated osteoclasts and the expression of NFATc1 and cathepsin K. In summary, Lrp5-overexpressing osteocytes markedly reduced tumor progression by regulating tumor-promoting, tumor-suppressing, and apoptosis-inducing genes.^24–27^

One notable feature of this study is the unique approach to inhibit tumor progression by activating Wnt signaling in osteocytes with overexpression of its receptor and cytoplasmic signal transducer. Since many lines of evidence report Wnt signaling as tumorigenic,^28^ the approach used herein attempted to inhibit tumor growth with a tumor-promoting agent or tumor-suppressing CM. Mature osteocytes express a high level of Sclerostin, a well-known marker of osteocyte differentiation.^8^ It has been reported that an elevated level of Sclerostin in peripheral blood is linked to poor prognosis in multiple myeloma patients.^17^ However, our results showed that A5 CM had a low level of Sclerostin and did not contribute to this issue. We showed that the silencing of Lrp5 in EO771 mammary tumor cells suppressed tumor-induced bone loss, while Lrp5 deletion in the conditional knockout mouse model stimulated bone degradation. This result indicates that systemic administration of a Wnt signaling inhibitor may interfere with the intrinsic antitumor ability of osteocytes in the bone microenvironment.

While two Wnt coreceptors, Lrp5 and Lrp6, are known to be involved in bone homeostasis,^29^ they played different roles in tumor progression in our study. Deletion of Lrp5 in tumor cells reduced mammary tumor growth, but deletion of Lrp6 did not. Additionally, Lrp5 shRNA-transfected osteocytes lost antitumor capability, but Lrp6 shRNA transfection did not affect the antitumor action of osteocytes. Considering these results collectively, we postulate that Lrp5 but not Lrp6 in osteocytes can enhance tumor suppression.

Cancer treatments include surgery, radiotherapy, chemotherapy, immunotherapy, hormone therapy, and stem cell transplantation,^30^ but injection of nonimmune differentiated cells or their CM is not a standard treatment. Ideally, Wnt signaling needs to be inhibited in tumor cells, but this study provided several lines of evidence indicating that it can be activated in osteocytes, with beneficial therapeutic outcomes. Although administration of Wnt inhibitors reduced tumor growth, they did not appreciably strengthen the antitumor effects of osteocytes. A chemotherapeutic drug may thus not be sufficient to regulate Wnt signaling in the tumor-osteocyte microenvironment. Notably, osteocytes presented intrinsic tumor-suppressive capabilities in breast cancer cells and tissues, while fibroblasts did not show antitumor capabilities. The present study indicates the possibility of osteocyte-based therapy—particularly to treat bone metastasis—in which osteocytes act as antitumor agents and orchestrate bone homeostasis. We found that overexpressing Lrp5 and constitutively activating β-catenin strengthened this tumor-suppressive capability.

The results herein shed light on Lrp5- and β-catenin-mediated antitumor actions of osteocytes, though several limitations should be noted. In this study, we employed C57BL/6 mice injected with EO771 mammary tumor cells and NOD/SCID mice injected with MDA-MB-231 human breast cancer cells. While this study employed a variety of cell models, not only breast cancer cell lines but also prostate cancer cells, primary human breast cancer cells and osteocytes, tumor-osteocyte interactions may depend heavily on the type of breast cancer cells. The antitumor capability of osteocytes may depend on their differentiation stage as well as the expression level of Lrp5. It is also necessary to examine whether any other cancer cells behave similarly to breast cancer cells.^31,32^ It has been reported that prostate cancer cells become invasive via interactions with osteocytes.^33^ We observed that osteocyte-derived CM increased the invasion of PC-3 prostate cancer cells. However, we also observed that CM from Lrp5-overexpressing osteocytes decreased the invasive behavior of these cells. It is recommended that the effect of osteocyte-derived CM be evaluated not only on breast cancer cells but also other cancer cells.

In conclusion, our study demonstrates that as the matrix-laden and most abundant cells in bone, osteocytes—and their CM—can inhibit tumor progression and bone loss and that this capability is enhanced by activating Wnt signaling via Lrp5 and β-catenin overexpression. Whereas much previous work has shown the importance of Wnt signaling in bone homeostasis and tumor progression, our work explores the tumor-suppressive role of Wnt signaling via tumor-osteocyte communication. The results herein point to the possibility of a novel osteocyte-based cancer therapy. Further studies are warranted to gain maximum benefit from local administration of engineered osteocytes or systemic administration of their CM to treat breast cancer-associated bone metastasis.

## Materials and methods

### Cell culture

EO771 mouse mammary tumor cells (CH3 BioSystems, Amherst, NY, USA),^34^ 4T1.2 mouse mammary tumor cells (obtained from Dr. R. Anderson at the Peter MacCallum Cancer Institute, Melbourne, Australia), and fibroblast cells (CRL3063; ATCC, Manassas, VA, USA) were cultured in DMEM. MDA-MB-231 breast cancer cells (ATCC), MLO-A5 and MLO-Y4 osteocyte-like cells (C57BL/6 background; obtained from Dr. L. Bonewald at Indiana University, IN, USA), and RAW264.7 preosteoclast cells (ATCC) were grown in αMEM. Human primary osteocytes (Celprogen, 36043-15) were maintained in human osteocyte primary cell culture complete growth medium (Celprogen, M36043-15S) and subcultured on extracellular matrix (Celprogen, E36043-15). PC-3 human prostate cancer cells (ATCC) were cultured in RPMI-1640 medium (Gibco, Carlsbad, CA, USA).^35^ Primary human breast cancer cells (ER^+^/PR^−^ 0514-15 cells and triple-negative 0514-21 cells) were grown as described previously.^36^ Culture media were supplemented with 10% fetal bovine serum and antibiotics (100 U·mL^−1^ penicillin and 100 μg·mL^−1^ streptomycin; Life Technologies, Grand Island, NY, USA), and cells were maintained at 37 °C in 5% CO_2_. Expression plasmids for Lrp5 (40 ng·μL^−1^) and β-catenin (40 ng·μL^−1^) were transfected into 2 × 10^6^ osteocytes overnight. After 1 d of incubation, the CM was ultracentrifuged to remove exosomes and condensed 10-fold by filtering (Amicon, Sigma, Saint Louis, MO, USA) with a molecular weight cutoff of 3 kD. Proteins from CM-treated cells were harvested 24 h after the beginning of incubation.

### EdU incorporation assay

Approximately 2 000 cells were seeded in 96-well plates on day 1. CM was added on day 2, and cell proliferation was examined using a fluorescence-based cell proliferation kit (Click-iT™ EdU Alexa Fluor™ 488 Imaging Kit; Thermo-Fisher, Waltham, MA, USA) on day 4. After fluorescent labeling, the number of fluorescently labeled cells was counted, and the ratio of the number of fluorescently labeled cells to the total number of cells was determined.^37^

### Invasion (as assessed by a Transwell assay) assay

The invasion capacity of cancer cells was determined using a 24-well plate, Transwell chambers (Thermo Fisher Scientific, Waltham, MA, USA) with an 8-μm pore size, and Matrigel (100 μg·mL^−1^). Approximately 5 × 10^4^ cells in 200 μL of serum-free DMEM were plated in the upper chambers, and 800 μL of CM was added to the lower chambers. After 48 h, the cells that had invaded to the lower side of the membrane were stained with crystal violet. At least five randomly selected images were acquired, and the average number of stained cells was determined.

### Two-dimensional motility assay

A scratch wound-healing motility assay was performed to evaluate 2-dimensional cell motility. Approximately 4 × 10^5^ cells were seeded in 12-well plates. After cell attachment, a plastic pipette tip was used to scratch a gap wound in the cell layer. Floating cells were removed, and CM was added. Images of the cell-free scratch wound zone were obtained via an inverted microscope at 0 h, and the areas newly occupied with cells were measured 24–48 h after wounding. The areas were quantified with ImageJ (National Institutes of Health, Bethesda, MD, USA).^38^

### Osteoclast differentiation assay

An osteoclast differentiation assay was conducted with RAW264.7 preosteoclast cells in 12-well plates. During the 6-day incubation of preosteoclast cells in medium containing 40 ng·mL^−1^ RANKL, the culture medium was exchanged once on day 4. Adherent cells were fixed and stained with a TRAP-staining kit (Sigma-Aldrich, Missouri, USA) according to the manufacturer’s instructions. TRAP-positive multinucleated (>3 nuclei) cells were identified as mature osteoclasts and counted.

### Western blot analysis and mass spectrometry

Western blot analysis was conducted using a previously described protocol.^39^ We used antibodies against ANXA1, β-catenin, caspase 3, Lrp5, Lrp6, Runx2, Sclerostin, Snail, TGFβ, NFATc1, cathepsin K (all from Cell Signaling, Danvers, MA, USA), DMP1, ANXA6, CXCL5 (all from Abcam, Cambridge, MA, USA), M-CSF, MMP9, OPN, TPM4 (all from Santa Cruz, Dallas, TX, USA), WISP1 (R&D systems, Minneapolis, MN, USA), β-actin (Sigma, Saint Louis, MO, USA), LIMA1, Trail (both from Novus, Centennial, CO, USA), p53, CXCL1 (both from Invitrogen, Carlsbad, California, USA), and DSP (ProteinTech, Rosemont, IL, USA). The expression levels of Sclerostin and Lrp5 in CM were detected by ELISA (My BioSource, San Diego, CA, USA). Proteins isolated from A5 osteocyte CM, Y4 osteocyte CM, and osteoclast control CM (RAW264.7 cells) were analyzed with an HF Hybrid Quadrupole Orbitrap mass spectrometer. Among the 549 identified proteins, 49 proteins had higher expression levels in A5 CM than in Y4 CM and control CM. Among these proteins, 11 (p53; SPARC = osteonectin; TPM1, TPM4 = tropomyosin 1 and 4; ANXA1, ANXA6 = annexin A1 and A6; FMOD = fibromodulin; OGN = osteoglycin; DSP = desmoplakin; AHNAK = desmoyokin; and LIMA1 = LIM domain actin-binding protein 1) were identified as potential tumor suppressors.

### Plasmid transfection, RNA interference, and cytokine analysis

To overexpress Lrp5 (#115907, Addgene, Watertown, MA, USA) or β-catenin (#31785, Addgene), A5 osteocytes or EO771 tumor cells were transfected with plasmids consisting of the coding sequence of the gene of interest, while a blank plasmid vector (FLAG-HA-pcDNA3.1; Addgene) was used as the control. A5 cells or EO771 cells were also treated with shRNA specific for Lrp5 (sc-149050-V, Santa Cruz), Lrp6 (sc-37234-V, Santa Cruz), or Runx2 (sc-37146-V, Santa Cruz), with GFP shRNA (sc-108084, Santa Cruz) used as the control. Cells were grown in a 10 cm plate and transfected with β-catenin plasmids or control plasmids using Lipofectamine^®^3000 (Thermo, L300015). First, plasmids/shRNAs were diluted in 200 μL of Opti-MEM, and 2 μL of P3000 was added for every 1 μg of DNA/shRNA. Then, 20 μL of Lipofectamine 3000 was mixed with 200 μL of Opti-MEM. Transfection was performed overnight, and stable shRNA transfectants were selected using puromycin (Sigma). In addition to shRNAs, siRNAs were employed for silencing of β-catenin and Trail, together with a nonspecific negative control siRNA (Silencer Select #1, Life Technologies; On-target Plus Nontargeting Pool, Dharmacon). Cells were transiently transfected with siRNAs with Lipofectamine RNAiMAX (Life Technologies). Twenty-four hours later, the medium was replaced with plain culture medium. The silencing efficiency was assessed by immunoblotting 24 h after transfection.^37^ We also employed a mouse XL cytokine array (R&D Systems) and determined the levels of 111 cytokines and chemokines in osteocyte-derived CM.

### 3D spheroid assay and ex vivo tissue assay

Cells were cultured in ultra-low attachment 96-well plates (S-BIO, New Hampshire, USA) at 1 × 10^4^ cells per well for EO771 cells and 5 × 10^3^ cells per well for A5 cells. Cells were imaged every 24 h, and the area was calculated with ImageJ. The usage of human breast cancer tissues in the ex vivo tissue assay was approved by the Indiana University Institutional Review Board. A sample (~1 g; ER/PR^+^, HER2^+^) received from the Simon Cancer Center Tissue Procurement Core was manually minced with a scalpel into small fragments (0.5–0.8 mm in length). These fragments were incubated in DMEM supplemented with 10% fetal bovine serum and antibiotics for 1 d. Osteocyte-derived CM was then added for 2 additional days, and a change in the fragment sizes was observed.^38^

### FRET imaging

To evaluate the tension force at focal adhesions and the migratory capacity of tumor cells in response to treatment with A5 CM and Runx2 shRNA, a plasmid expressing a vinculin tension sensor (VinTS, #26019, Addgene) was transfected. Fluorescence lifetime images were acquired with a custom-made microscope based on a laser scanning confocal microscope (FluoView 1000, Olympus; Center Valley, PA, USA) using previously described procedures.^39^ A picosecond pulsed laser with a wavelength of 450 nm was coupled to the laser scanning module. All signals were recorded in the time-correlated single-photon-counting mode with a data acquisition board (TimeHarp 260, Picoquant; Berlin, Germany). The FRET efficiency of the TS module was calculated based on the lifetime of the donor molecule. Of note, an increase in the tension force of the vinculin sensor implies an increase in the fluorescence lifetime.

### Animal models

The procedures for animal experiments were approved by the Indiana University Animal Care and Use Committee and complied with the Guiding Principles in the Care and Use of Animals endorsed by the American Physiological Society. C57BL/6 mice lacking Lrp5 in osteocytes (Dmp1-Cre; Lrp5f/f) were obtained by breeding Dmp1-Cre transgenic mice with Lrp5 floxed mice, both of which have been described earlier.^40^ Mice were housed five per cage and provided with mouse chow and water ad libitum. In the mouse model of mammary tumors, female C57BL/6 mice (~8 weeks; Envigo RMS, Inc., Indianapolis, IN, USA) and NOD/SCID mice (~8 weeks; The Jackson Laboratory, Bar Harbor, ME, USA) received subcutaneous injections of EO771 cells and MDA-MB-231 cells (3.0 × 10^5^ cells in 50 μL of PBS), respectively, into the seventh mammary fat pad on day 1.^41^ We also conducted a proof-of-principle study in which osteocytes were injected into mammary fat pads. This proof-of-principle model system was employed as an early-stage evaluation of the therapeutic feasibility of osteocytes. In the treatment groups, A5 osteocytes (1.5 × 10^5^ cells) were coinjected with EO771 cells or MDA-MB-231 cells on day 1, and osteocyte-derived CM was injected into the intraperitoneal cavity from day 2 to day 18. The animals were sacrificed on day 18, and the weight of each tumor was measured. In the mouse model of osteolysis, ten female C57BL/6 mice per group were injected in the right tibia with EO771 cells (3.0 × 10^5^ cells in 20 μL PBS) on day 1. A5 osteocytes (1.5 × 10^5^ cells) were coinjected with EO771 cells into the proximal tibia on day 1. Intramuscular injection of osteocyte-derived CM into the proximal tibia was conducted from day 2 to day 18. EO771 cells were transfected with shRNA specific for Lrp5, Lrp6, or Runx2. Osteocytes were transfected with shRNA specific for Lrp5 or Lrp6 or with expression plasmids for Lrp5 or β-catenin.

To evaluate the effects of CMs on tumor invasion, an in vivo extravasation assay was conducted. Female C57BL/6 mice (5 mice per group) were injected with 50 μL of fluorescently labeled EO771 cells (1.0 × 10^6^ cells) via the lateral tail vein. Fluorescently labeled EO771 cells were prepared by culture with a green fluorescent dye (#4705, Sartorius, Gottingen, Germany) for 20 min at 37 °C. Cells were then centrifuged at 1 000 r·min^−1^ for 5 min to harvest the pellet. The pellet was resuspended in PBS (placebo group) or osteocyte-derived CM (A5 CM group). Mice were sacrificed after 48 h for histological identification of extravasated tumor cells in the lung.

### X-ray and MR imaging

Whole-body X-ray imaging was performed using a Faxitron radiographic system (Faxitron X-ray Co., Tucson, AZ, USA).^42^ Tibial integrity was scored in a blinded manner on a scale of 0–3, as follows: 0 = normal with no indication of a tumor, 1 = clear bone boundary with slight periosteal proliferation, 2 = bone damage and moderate periosteal proliferation, and 3 = severe bone erosion.^43^ MR imaging was conducted with a Bruker 7T 70/30 USR system (Bruker BioSpin Co., Billerica, MA, USA).^44^ We employed the Turbo RARE sequence for high resolution (T2-weighted imaging) with the Bruker interface (Paravision V6.0.2).

### μCT imaging and histological analysis

Tibiae were harvested for μCT imaging and histological analysis. Microcomputed tomography was performed using a Skyscan 1172 instrument (Bruker-MicroCT, Kontich, Belgium). Using manufacturer-provided software, scans were performed at a pixel size of 8.99 μm, and the images were reconstructed (nRecon v1.6.9.18) and analyzed (CTan v1.13). For histological analysis, H&E staining was conducted as described previously,^38^ and immunohistochemistry was performed using a previously described protocol.^45^

### Statistical analysis

For cell-based experiments, three or four independent experiments were conducted, and data are expressed as the mean ± S.D. values. For animal experiments, the sample size in the mouse model was chosen to achieve a power of 80% with a significance level of 0.05. The primary experimental outcome was tumor weight for the mammary fat pad experiment and the BV/TV for the tibia experiment. The secondary experimental outcome was tumor size for the mammary fat pad experiment and the Tb.N for the tibia experiment. Statistical significance was evaluated using one-way analysis of variance (ANOVA). Post hoc statistical comparisons with control groups were performed using the Bonferroni correction with statistical significance assumed for *P* < 0.05. A nonparametric Kolmogorov–Smirnov test was applied to compare cell aspect ratios. The single and double asterisks in the figures indicate *P* < 0.05 and *P* < 0.01, respectively.

## Supplementary information

Supplmental information
