# Supplementary material for: Overexpression of Lrp5 enhanced the anti-breast cancer effects of osteocytes in bone
Source: Bone Res. 2021 Jul 6;9:32. doi: 10.1038/s41413-021-00152-2 (PMC8260600; doi:10.1038/s41413-021-00152-2)

Supplementary Information

Suppl. Figure 1. Effects of MLO-Y4 pre-osteocyte (Y4)-derived CM and fibroblast-derived CM on cellular behaviors of EO771 and 4T1.2 mammary tumor cells. Of note, CN = control (plain medium), CM = conditioned medium, pL5 = Lrp5 plasmids, siL5 = Lrp5 siRNA, and Fib = fibroblasts. The single and double asterisks indicate p < 0.05 and p < 0.01, respectively. Scale bar: 200 μm. (a) Significant reduction of cellular migration by Y4 CM and Lrp5-overexpressing Y4 CM in EO771 and 4T1.2 cells. (b) Effects of Y4 CM with and without Lrp5 siRNA treatment in EdU-based proliferation and migration of EO771 cells. (c&d) Promotion of the proliferation and migration by fibroblast-derived CM with and without Lrp5 overexpression in EO771 and 4T1.2 cells. Of note, fibroblasts were employed as a negative control in this study. (e) Growth promotion of *ex vivo* breast cancer tissue fragments by fibroblast-derived CM with and without Lrp5 overexpression.

Suppl. Figure 2. Effects of primary human osteocyte (huO)-derived conditioned medium on MDA-MB-231 breast cancer cells, and PC-3 human prostate cancer cells. Of note, huO = human osteocytes, CN = control (plain medium), CM = conditioned medium, shL5 = Lrp5 shRNA, and pL5 = Lrp5 plasmids. The single and double asterisks indicate p < 0.05 and p < 0.01, respectively. Scale bar: 200 μm. (a) Reduction in cellular proliferation, invasion, and migration of MDA-MB-231 cells by human osteocyte-derived CM with and without Lrp5 overexpression. (b&c) Inhibitory effect of human osteocyte-derived CM on EdU-based cellular proliferation and invasion in PC-3 prostate cancer cells.

Suppl. Figure 3. Shrinkage of mammary tumor in C57BL/6 and NOD/Scid mice by the co-injection of A5 osteocytes, and the effects of Lrp6 silencing in EO771 tumor cells and A5 osteocytes on mammary tumors in C57BL/6 mice.. Of note, pL5 = Lrp5 plasmids, shL5 = Lrp5 shRNA, shCN = control shRNA, shL5 = Lrp5 shRNA, and slL6 = Lrp6 shRNA. The single and double asterisks indicate $p < 0.05$ and $p < 0.01$, respectively. Scale bar: 1 cm. (a) Schematic illustration of C57BL/6 female, showing the inoculation

of EO771 mammary tumor cells with and without the co-injection of A5 osteocytes to the mammary fat pad. The results showed a significant reduction in tumor progression by the co-injection of A5 osteocytes (N = 10). (b-d) Effects of osteocyte inoculation with and without Lrp5 shRNA or Lrp5 plasmids on the tumor size and weight in NOD/Scid mice (N = 10). The pink ribbon indicates no detectable tumor. The histological section shows GFP-labeled osteocytes in the mammary tumor. (e&f) No significant effect of Lrp6 silencing in EO771 cells (N = 10). (g&h) Differential effects of the silencing of Lrp5 and Lrp6 in A5 osteocytes. Lrp5 silencing suppressed the anti-tumor capability with A5 osteocytes, but Lrp6 silencing did not significantly alter it (N = 10).

Suppl. Figure 4. Protection of tumor-induced osteolysis in the tibia in C57BL/6 mice by the co-injection of A5 osteocytes, and by Lrp5 silencing in EO771 tumor cells. Of note, shCN = control shRNA, shL5 = Lrp5 shRNA, CN = normal control, WT = wildtype, and KO = knockout. The single and double asterisks indicate $p < 0.05$ and $p < 0.01$, respectively. (a) H&E-stained images of the proximal tibia for the placebo, A5 osteocyte-injected group, and Lrp5shRNA-treated osteocyte group. The relative tumor area in the proximal tibia is presented (N = 8). Scale bar: 100 μm. (b) Effects by silencing of Lrp5 in EO771 on the tumor size and weight (N = 8). Scale bar: 500 mm. (c) Reduction in tibial osteolysis by silencing of Lrp5 in EO771 tumor cells (N = 8). Of note, BV/TV = bone volume normalized by total volume, BMD = bone mineral density, Tb.n = trabecular number, and Tb.s = trabecular separation. Scale bar: 500 μm. (d) Reduction in BV/TV in the tibia caused by Lrp5 deletion in the osteocytes (N = 8). Scale bar: 500 μm. (e) Tumor-induced decrease of BV/TV in the tibia was more severe than the decrease caused by Lrp5 deletion.

Suppl. Figure 5. Effects of Lrp5 plasmids and BML284, an activator of Wnt signaling on tumor progression. Of note, CN = control (plain medium), CM = conditioned medium, pL5 = Lrp5 plasmids, siβ or siβ-c = β-catenin siRNA, and BML = BML284. The double asterisk indicates $p < 0.01$. (a) Relative

intensity levels of tumor-promoting cytokines/chemokines in A5 osteocyte-derived CM with and without Lrp5 overexpression. (b) Elevation of apoptosis-linked genes (CYCS, HIF1α, and APT1) in EO771 cells of β-catenin-overexpressing osteocyte-derived CM. (c) Suppression of the inhibitory effect of Lrp5-overexpressing A5 CM by RNA interference with β-catenin siRNA on EdU-based cellular proliferation and invasion in EO771 breast cancer cells. Scale bar: 200 μm. (d) Levels of Sclerostin and Lrp5 in A5 CM with and without β-catenin-overexpression and BML284 treatment. (e) Shrinkage of *ex vivo* breast cancer tissue fragments by BML284-treated, osteocyte-derived CM. Scale bar: 200 μm. (f) Downregulation of Lrp5, MMP9, Runx2, Snail, and TGFβ in EO771 tumor cells in response to BML284-treated, osteocyte-derived CM. (g) Reduction in the number of green-fluorescently-labeled EO771 mammary tumor cells in the lung by BML284 co-injection in the extravasation assay. (h) Reduction in mammary tumors in CL57BL/6 mice by systemic administration of BML284-treated, osteocyte-derived CM. Scale bar: 1 cm.

Suppl. Figure 6. Gene regulation in β-catenin-overexpressing A5 osteocytes and EO771 mammary tumor cells, and effects of β-catenin-overexpressing in A5 osteocytes on tibia tumor progression. Of note, NC = non-specific control vector, pβ-cat = β-catenin plasmids, CN = control (plain medium), CM = conditioned medium, and siT = Trail siRNA. (a) Expression of tumor-suppressing genes (TPM4, ANXA1, ANXA6, LIMA1, p53, and DSP) in β-catenin overexpressing osteocytes. (b) Expression of CXCL1, CXCL5, WISP1, OPN, and M-CSF in EO771 cells in response to β-catenin overexpressing osteocyte-derived CM. (c) Expression of tumor-promoting genes (Lrp5, MMP9, Runx2, and Snail) in EO771 cells in response to TGFβ and CXCL5. (d) Expression of Lrp5, MMP9, Runx2, TGFβ, Snail, and cleaved caspase 3 in EO771 cells in response to TPM4, ANXA6, and Trial. (e) Trail expression in A5 osteocytes in response to β-catenin plasmids with and without Trail siRNA. (f) Inhibitory effect of A5 CM with and without β-catenin-overexpression on cellular proliferation and invasion in EO771 breast cancer cells. Of note, RNA interference with Trail siRNA partially suppressed the inhibitory effect with the overexpression of β-

catenin. Scale bar: 200 μm. (g) Reduction of bone degradation in the tibia in C57BL/6 mice by the systemic administration of β-catenin-overexpressing osteocyte-derived CM. Scale bar: 1 mm.

Suppl. Figure 7. Involvement of Runx2 in the anti-tumor capability of A5 osteocytes. Of note, CN = control (plain medium), CM = conditioned medium, shCN = control shRNA, and shR2 = Runx2 shRNA. The single and double asterisks indicate $p < 0.05$ and $p < 0.01$, respectively. (a) Reduction in Runx2 and MMP9 in 2 sources of primary human breast cancer cells by A5 osteocyte CM. (b) Reduction in FRET lifetime (weakening molecular force) in EO771 tumor cells by Runx2 silencing in A5 osteocytes. Scale bar: 20 μm. (c) Shrinkage of the mammary tumor in C57BL/6 mice by silencing Runx2 in EO771 tumor cells. Scale bar: 1 cm.

Suppl. Figure 1

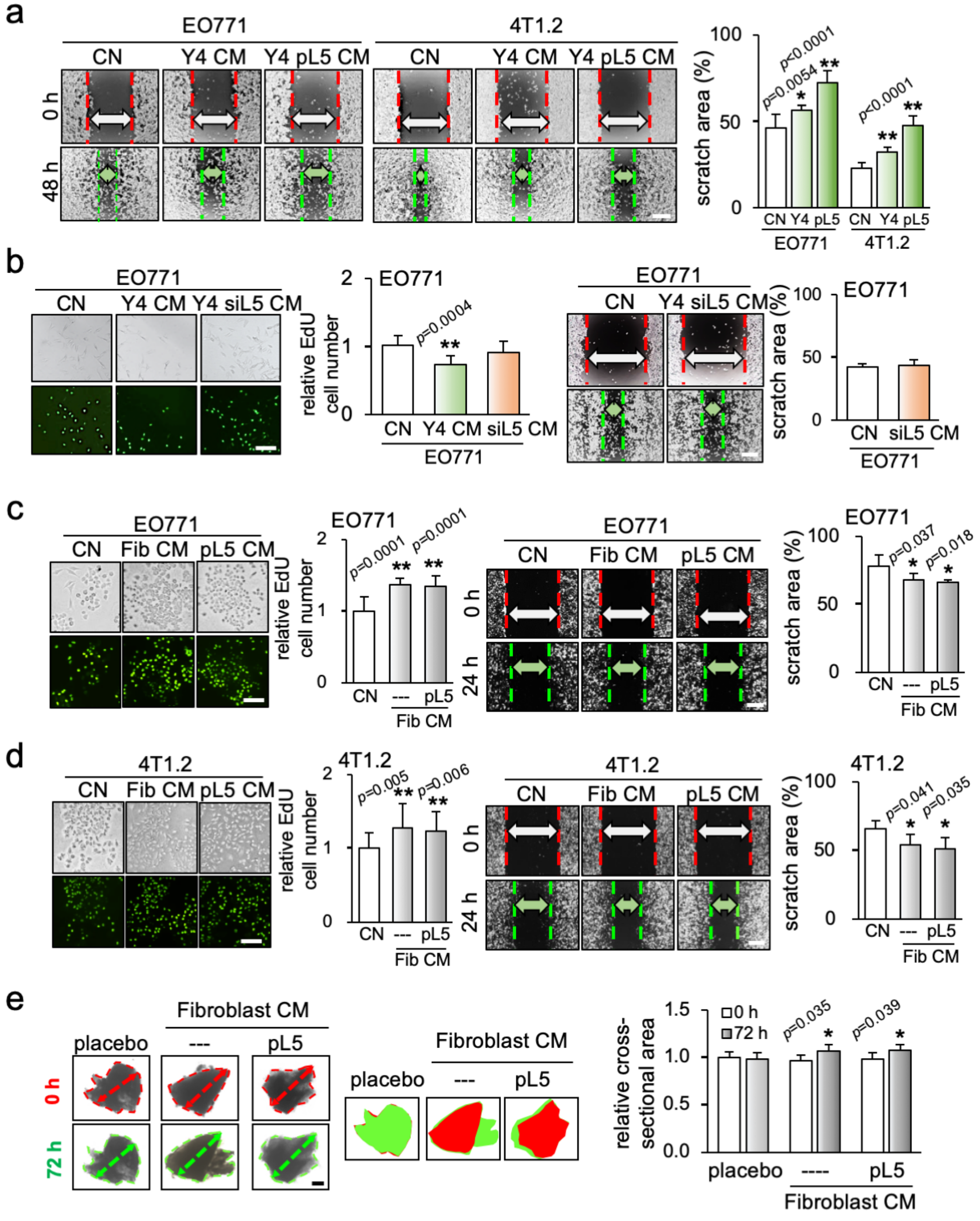

Suppl. Figure 2

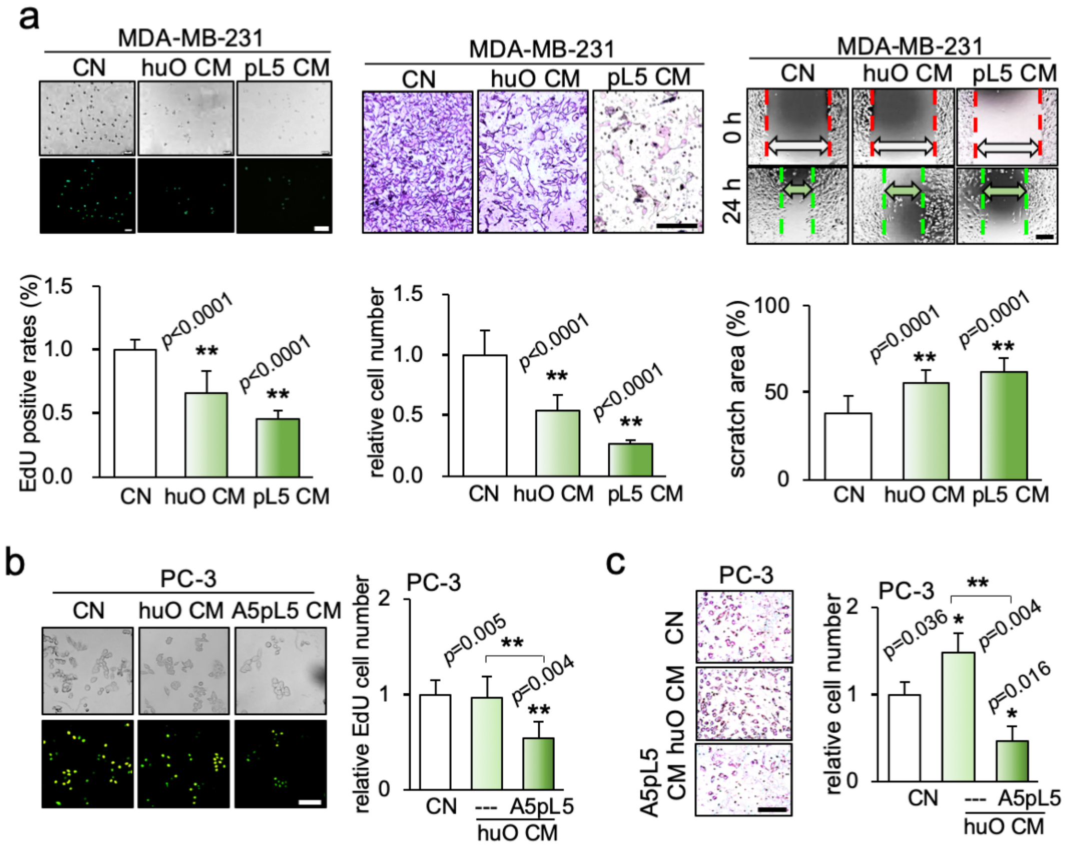

Suppl. Figure 3

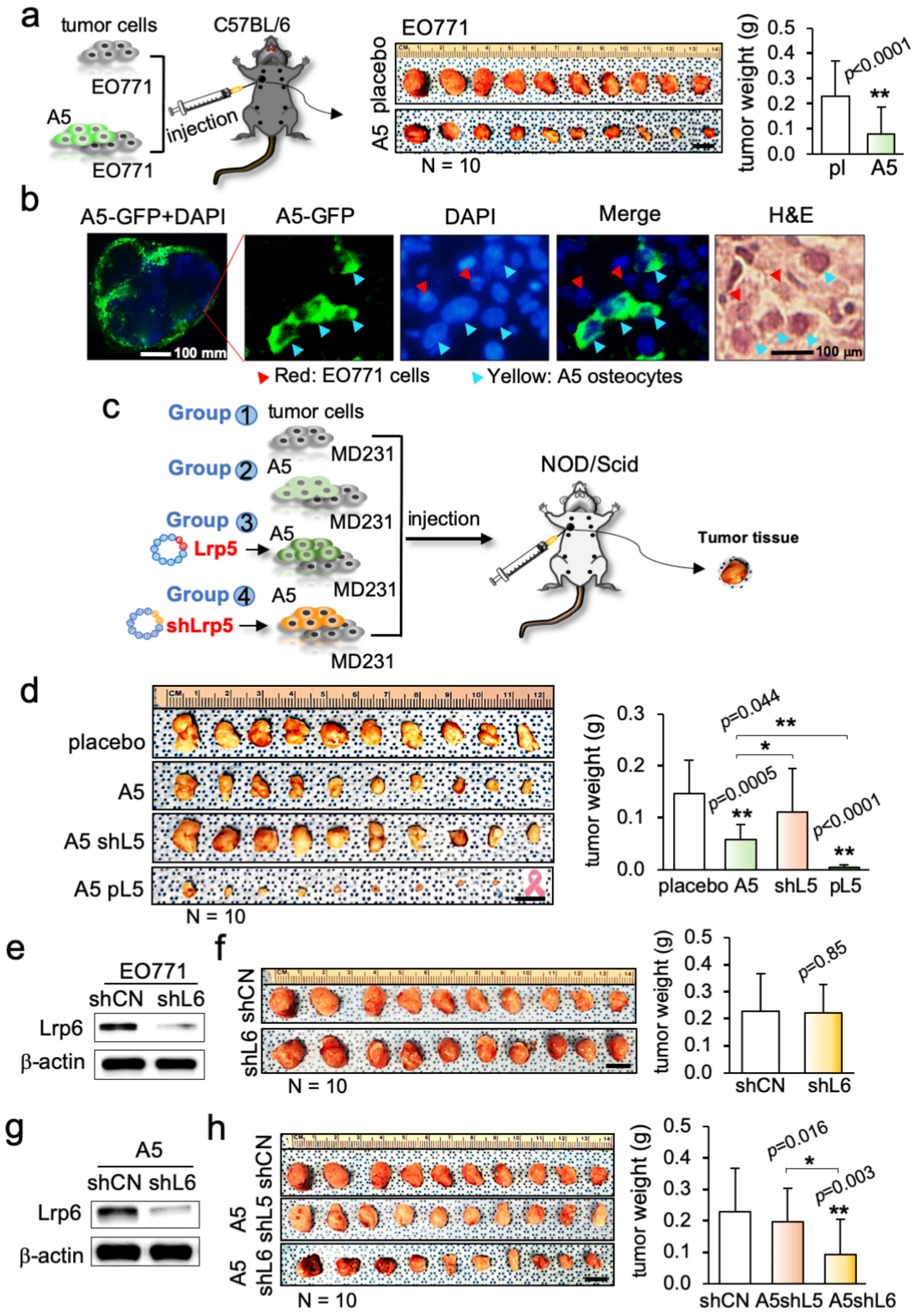

Suppl. Figure 4

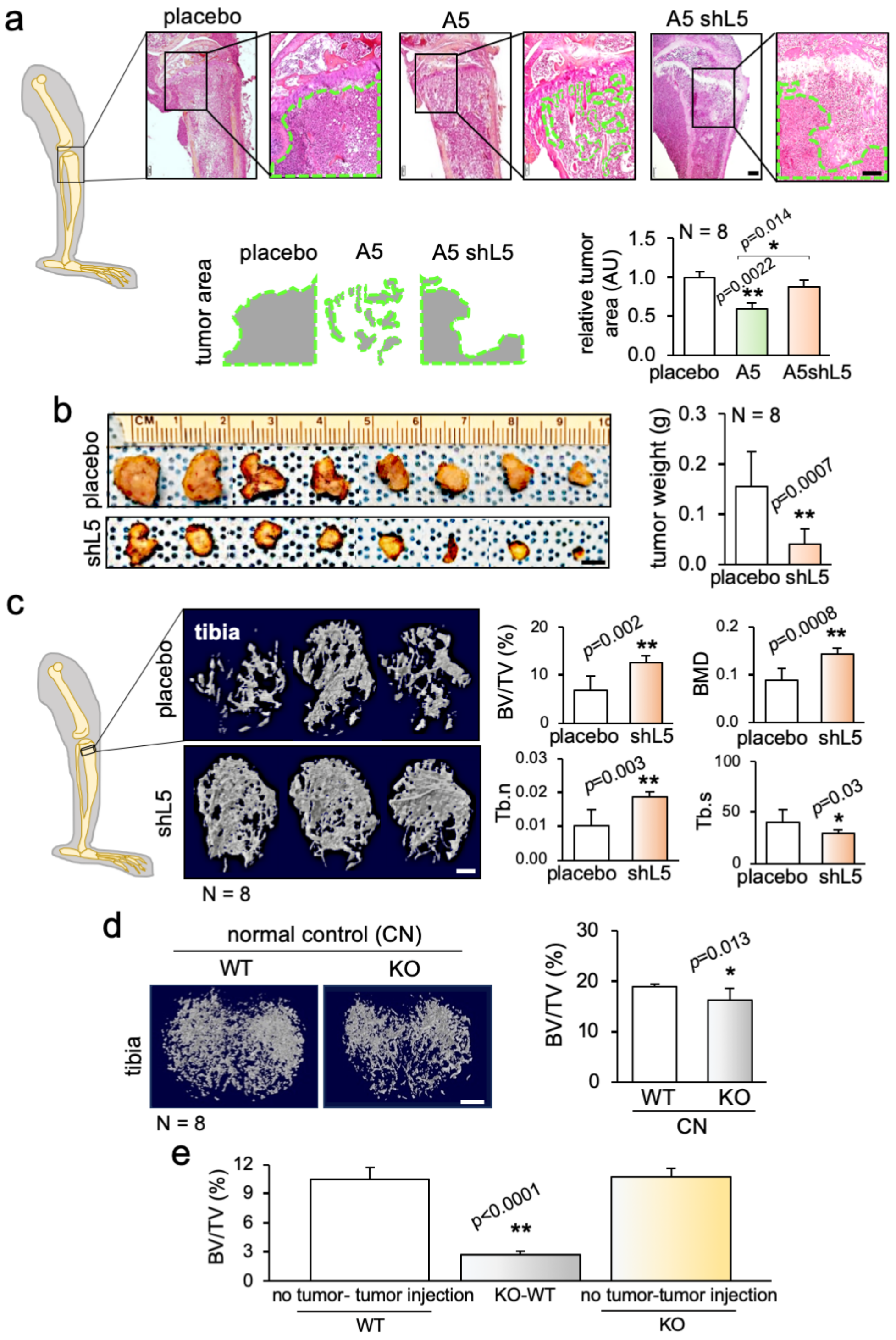

Suppl. Figure 5

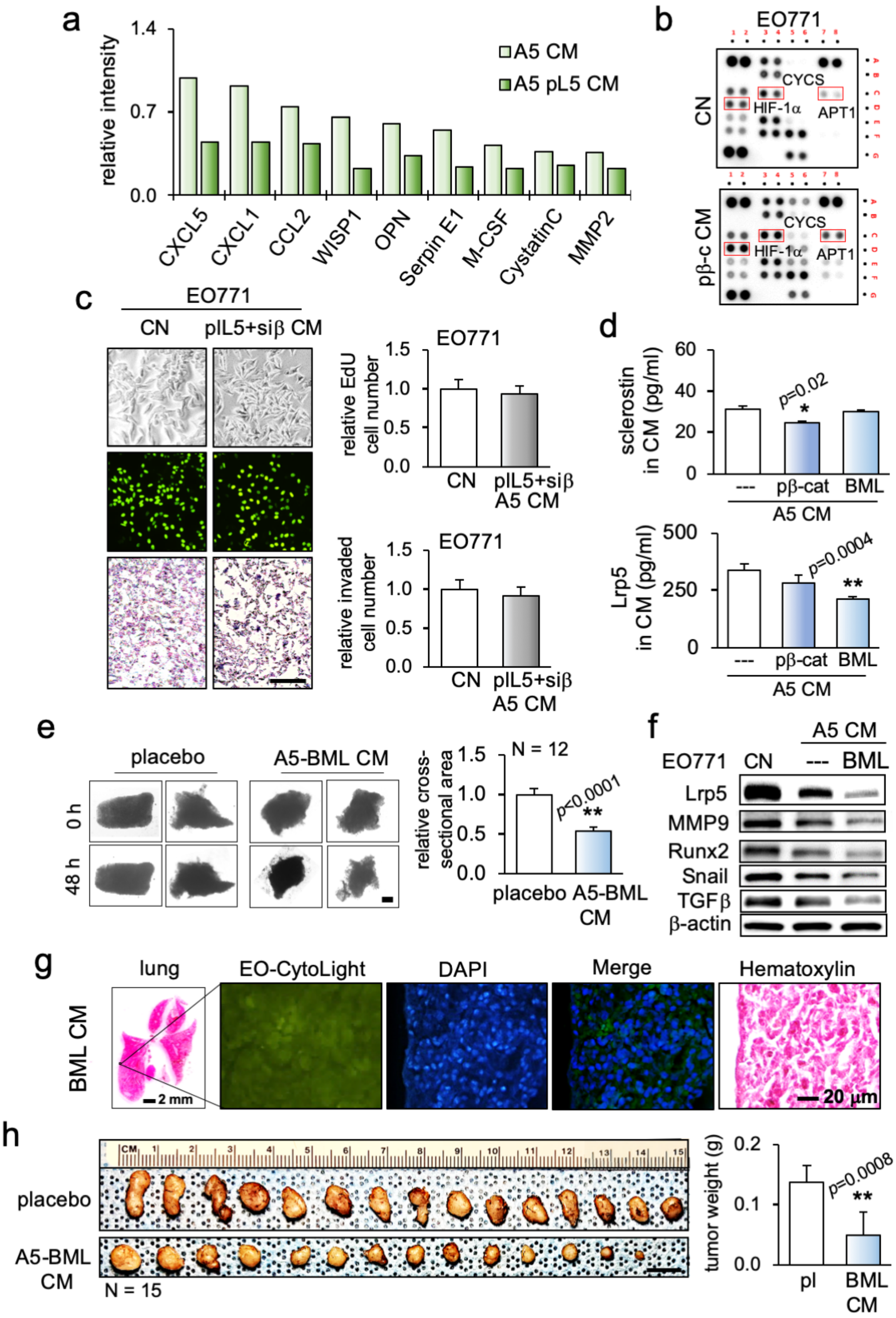

Suppl. Figure 6

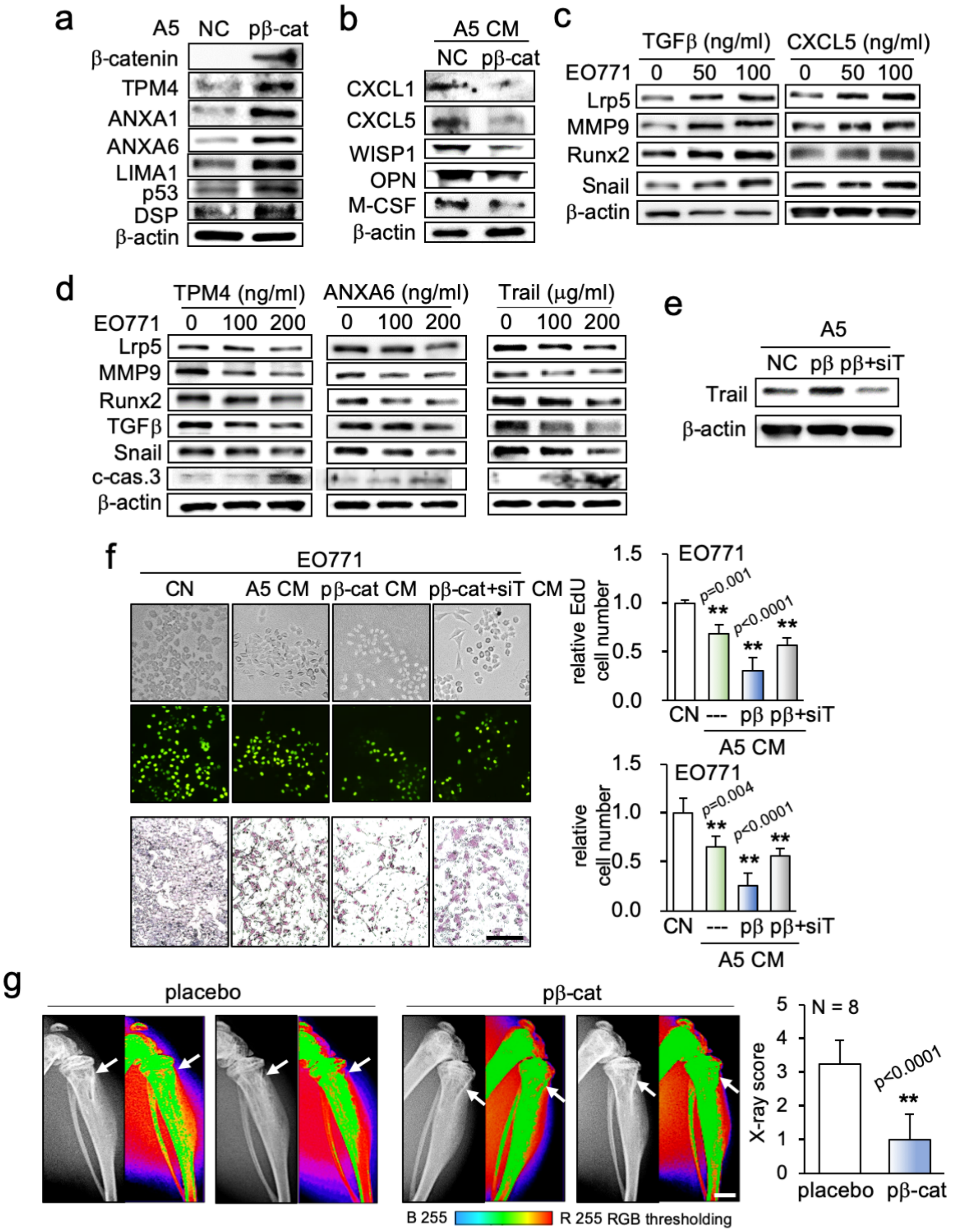

Suppl. Figure 7

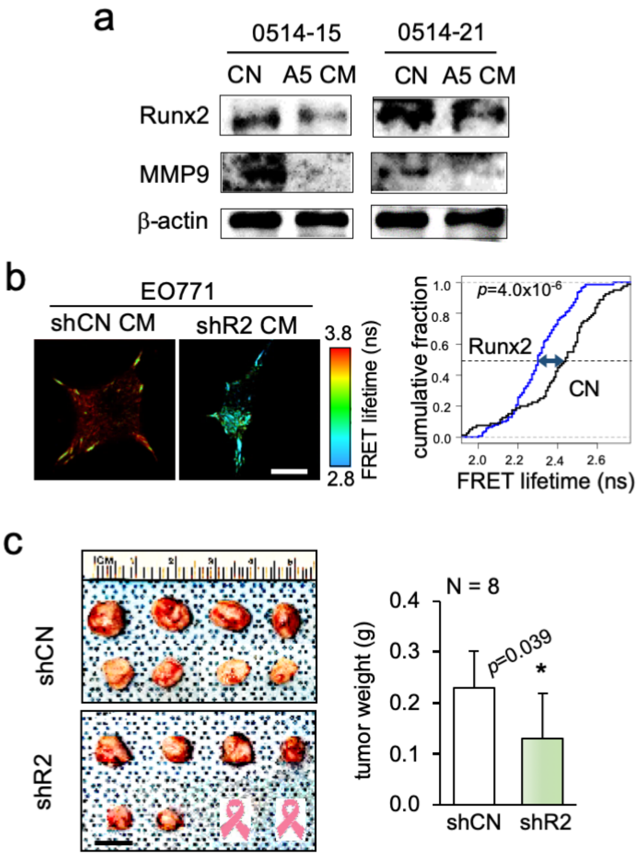

Supplement: Supplementary file 1 — Supplmental information [file 41413_2021_152_MOESM1_ESM.pdf]
